# Taking More Society for Academic Emergency Medicine Practice Tests Does Not Lead to Improved National EM-M4 Exam Scores

**DOI:** 10.5811/westjem.2022.12.57683

**Published:** 2023-01-16

**Authors:** David J. Story, Hong Gao, Andrea L. Vallevand, David Manthey

**Affiliations:** * Wake Forest Baptist Medical Center, Department of Emergency Medicine, Winston-Salem, North Carolina; † Wake Forest School of Medicine, Office of Undergraduate Medical Education, Winston-Salem, North Carolina; ‡ Wake Forest School of Medicine, Department of Emergency Medicine, Winston-Salem, North Carolina

## Abstract

**Introduction:**

Emergency medicine (EM) is a required clerkship for third-year medical students, and an elective EM acting internship (AI) is available to fourth-year students at our institution. The Society for Academic Emergency Medicine’s (SAEM) National Emergency Medicine M4 Examination (EM-M4) is administered to students at the end of the EM AI experience. To prepare for the exam, students gain access to 23 practice tests available from SAEM. In this study we investigate the correlation between the number of practice tests taken and EM-M4 performance.

**Methods:**

We collected data for EM-M4 and the US Medical Licensing Exam (USMLE) Step 2 Clinical Knowledge (CK) from students completing a MS4 EM clerkship in consecutive medical school classes from 2014–2017 at a private medical school. In addition, we collected data during the clerkship on the number of practice exams taken and whether a comprehensive practice exam was taken. We analyzed the study population three ways to determine whether the number of practice tests impacted final exam results: a binary distribution (1–11 or 12–23 tests taken); quaternary distribution (1–6, 7–12, 13–18, or 19–23 tests taken); and individual test variability (1,2,3,…22,23 tests taken). Complete data for 147 students was used for data analysis.

**Results:**

The EM-M4 showed moderate (r = 0.49) correlations with USMLE Step 2 CK. There was no significant difference in EM-M4 performance in the binary analysis (*P* ≤ 0.09), the quaternary analysis (*P* ≤ 0.09), or the continuous variable analysis (*P* ≤ 0.52). Inclusion of a comprehensive practice test also did not correlate with EM-M4 performance (*P* ≤ 0.78).

**Conclusion:**

Degree of utilization of SAEM practice tests did not seem to correlate with performance on the EM-M4 examination at our institution. This could be due to many factors including that the question bank is composed of items that had poor item discrimination, possible inadequate coverage of EM curriculum, and/or use of alternative study methods. While further investigation is needed, if our conclusions prove generalizable, then using the SAEM practice tests is an extraneous cognitive load from a modality without proven benefit.

## INTRODUCTION

A recent survey indicates that more than half of the medical schools in the United States require emergency medicine (EM) clerkships in their undergraduate medical curricula.^1^ There are currently two validated national EM exams (National Board of Medical Examiners [NBME] and Society for Academic Emergency Medicine [SAEM]), each with multiple forms available to clerkship directors for use in assessing the knowledge of their students. At our institution, an acting internship (AI) in EM for fourth-year medical students (MS4) has been offered for several years using the SAEM M4 National Exam as the end-of-rotation testing modality.

The first national EM exam was developed in 2010, when the Clerkship Directors in Emergency Medicine (CDEM) membership appointed a task force with the goal of developing an MS4-level test suitable for “high-stakes” evaluation of students rotating in EM.^2^ The task force based the content of the examination on the EM MS4 curriculum guide that was defined by CDEM in 2006 and revised in 2010.^3^,^4^ CDEM had previously created a question bank in 2005 consisting of 565 items that were divided into 22 different system-based tests and two comprehensive tests.^5^ These tests were evaluated by members of the task force in search of questions that fit the curriculum, showed high reliability and validity, and adhered to NBME item-writing guidelines.

Thirteen additional questions were written to assess the entirety of the curriculum. The result was a 50-question test (EM-M4) aimed at evaluating a student’s knowledge of the topics that should be gained in a fourth-year EM rotation.^2^ The exam began being offered in 2011 and became the testing modality for our EM AI. Previous studies on the SAEM final tests found they are moderately correlated with the US Medical Licensing Examination (USMLE) Step 1, and USMLE Step 2 Clinical Knowledge (CK).^6^ The unselected questions from the original question bank has been preserved and promoted as a study tool at www.saemtests.org. We provided students access to this question bank during the EM AI.

Testing can have multiple learner benefits with respect to memory retrieval and long-term retention.^7^–^9^ The format of the tests requires asking the right questions in the right format.^9^ As the SAEM practice tests were made of questions not selected for the EM-M4 exam there may be a concern as to the quality of the questions.^2^ As well, 13 additional questions had to be written to cover the entire curriculum, which suggests the practice test questions would not cover the entire curriculum.^2^

As educators, we want to promote techniques and sources that will benefit the student in the goal of learning the material critical for the successful understanding of the subject matter. The promotion and usage of the SAEM question bank as a study tool for students led to this study investigating the following questions:

1. Do students who take a higher number of practice tests have a significantly higher performance on the EM-M4 examination?
2. Does including a comprehensive practice test impact EM-M4 examination performance?

## MATERIALS AND METHODS

### Setting

The Wake Forest School of Medicine program is four years in duration. Students are required to pass the USMLE Step 1 examination to be promoted into the clinical program (Years 3 and 4). Eight mandatory clerkships, including a four-week EM clerkship, comprise Year 3. An AI in EM is available during Year 4 as an elective, and the SAEM EM-M4 exam is required of all students on the last day of the rotation. Students are given access to the SAEM practice exams and are provided a copy of the study guide “First Aid for the EM Clerkship” for use during the EM AI.

**Population Health Research Capsule**

What do we already know about this issue?

*To our knowledge, no prior studies have investigated the relationship between the number of SAEM practice exams attempted and National EM M4 exam performance.*

What was the research question?

*This study investigates whether there is a correlation between the number of SAEM practice exams taken and National EM M4 exam score.*

What was the major finding of the study?

*Taking a higher number of SAEM practice exams did not lead to improved National EM M4 exam performance (p < 0.09).*

How does this improve population health?

*Educators should promote study modalities with proven benefit to reduce extraneous cognitive load for medical students with a finite amount of study time.*

### Participants

The study group was composed of MS4 students enrolled in the EM AI representing three consecutive medical school classes from 2014–2017 who took at least one practice test.

### Procedures

The USMLE Step 2 CK (hereafter referred to as Step 2 CK) and EM-M4 examination scores were collected for all participants and subsequently de-identified for statistical analysis. We recorded and incorporated Step 2 CK scores into the analysis as a comparative variable. De-identified data was also collected on the total number of SAEM practice tests attempted and whether a SAEM comprehensive practice test was completed. We obtained this data directly from the www.saemtests.org website.

### Statistical Analysis

We analyzed Step 2 CK scores, number of SAEM practice tests taken, and EM-M4 scores using descriptive statistics. Completion of a SAEM comprehensive test was analyzed using frequency statistics. We investigated correlational analysis between EM-M4 and Step 2 CK performances using Pearson’s r.

An analysis of covariance (ANCOVA) explored Step 2 CK as a moderator on EM-M4 performance based on the number of practice tests taken. We analyzed the cohort three ways: continuous independent data (1–23 individually); binary distribution (1–11 vs 12–23 tests taken); and quaternary distribution (1–6, 7–12. 13–18 and 19–23 tests taken). We conducted a subgroup analysis of EM-M4 performance based on whether a comprehensive practice test was completed (yes or no). Test statistics and adjustments in EM-M4 mean scores from pre- to post-ANCOVA are reported.

The Institutional Review Board of Wake Forest School of Medicine approved this study.

## RESULTS

### Participants

We collected data from 147 students from three consecutive medical school classes (2014: n = 54 [37%]; 2015: n = 47 [32%]; 2016: n = 46 31%]).

### Descriptive Statistics

The USMLE Step 2 CK and EM-M4 scores and number of practice tests taken (mean, standard deviation, minimum, maximum and 95% confidence interval are presented in Table 1. Frequency analyses revealed 82/147 (55.8%) of students completed at least one comprehensive practice test in preparation for the EM-M4 examination. There was a significant relationship between Step 2 CK and EM-M4 scores, r = .46, *P* ≤ 0.01. Of note, the Step 2 CK and EM-M4 mean scores of the study cohort were slightly higher than the national average, but within the standard deviation of the exams.^11^,^12^

#### Number of practice tests taken (2 groups)

The covariate, Step 2 CK, was significantly related to performance on the EM-M4 examination F[1,144] = 36.927, *P* ≤ 0.001. There was no statistically significant effect on the number of practice tests taken, after controlling for Step 2 CK, F[1,144] = 2.856, *P* ≤ 0.09. The adjustments from pre-to-post ANCOVA mean for the EM-M4 variable are presented in Table 2.

#### Number of practice tests taken (four groups)

The covariate, Step 2 CK, was significantly related to performance on the EM-M4 examination F[1,142] = 31.165, *P* ≤ 0.001. There was no statistically significant effect on the number of practice tests taken, after controlling for Step 2 CK, F[3,142] = 2.206, *P* ≤ 0.09 (Table 3).

#### Number of practice tests taken as a continuous variable (23 groups)

The covariate, Step 2 CK, was significantly related to performance on the EM-M4 examination F[1,123] = 29.790, *P* ≤ 0.001. There was no statistically significant effect on the number of practice tests taken between the 23 groups, after controlling for Step 2 CK, F[22,123] = 0.961, *P* ≤ 0.52 (Table 4).

#### Whether a comprehensive test was taken (yes or no)

The covariate, Step 2 CK, was significantly related to performance on the EM-M4 examination F[1,144] = 37.329, *P* ≤ 0.001. There was no statistically significant effect on the number of practice tests taken, after controlling for Step 2 CK, F[1,144] = 0.081, *P* ≤ 0.78 (Table 5).

## DISCUSSION

In this study we set out to investigate whether the usage of the SAEM question bank correlates with improved scores on the EM-M4 exam. Our data indicates that taking SAEM practice tests, both system-based and comprehensive, did not significantly improve EM-M4 test performance when adjusting for Step 2 CK performance. There was a moderate correlation between Step 2 CK and EM-M4 scores similar to those observed by Lawson and colleagues.^6^

The EM-M4 exam is reflective of what a fourth-year medical student should learn over the course of a clinical rotation in EM. If we are recommending that students use the question bank as a method of learning the required material, and thus preparing for the exam, then it is important for that process to be a meaningful method for gaining knowledge. Because there is a finite amount of time for extraneous cognitive load during a clinical rotation, it is imperative that recommended study materials be high yield for students. Based on our results, students may not be gaining the intended benefit of the practice tests, and thus spending hours using a study modality with limited value.

This lack of benefit is contrary to current theory and what has been found in prior studies on practice tests, which show that repeated study and regular testing result in better organization of knowledge and improve application of knowledge to new contexts.^7^–^9^,^12^,^13^ Although we have no way of defining causation for this correlation, one explanation might be that the questions in the SAEM practice tests were not accepted for inclusion in the EM-M4 examination. Reasons for exclusion included poor item discrimination values, questions not addressing the National EM M4 curriculum, and item-writing flaws.^2^

Use of practice bank questions that were analytically removed in the creation of an examination may not be the best study preparation for that exam. Additionally, 13 additional questions had to be written because the available practice tests did not cover all the material on the tests.^2^ It is unknown whether additional questions covering the missing topics were written and added to the practice tests, but if not then a gap in the curriculum would still exist within the practice-test question bank.

Other explanations may include that the EM test is developed on a defined curriculum which is supported by an online website specifically for that curriculum. As such the use of additional materials may not add as much as in broader scenarios. Perhaps students not utilizing the practice tests prepared in other just as beneficial ways, negating a difference in taking more tests but not the importance of taking a practice test to identify where to study. As we could not look at zero tests versus one test in our cohort, we do not know that doing at least one test made a significant difference. Finally, taking a test versus taking a test to promote recognition, recall, and understanding are two different approaches and we have no way of knowing the mindset of the student taking a given practice test.

Additionally, we chose to investigate student utility of taking a comprehensive practice test and whether EM-M4 performance was impacted. Taking a comprehensive practice test lends itself to identifying an area of weakness, while the system-specific practice tests may address filling a knowledge gap. Unfortunately, only a raw score is provided to the student after completing a comprehensive test. Without a breakdown of performance within certain areas or topics within emergency medicine, the student does not receive any guidance regarding where to focus future study. This may limit the value of the SAEM practice comprehensive tests in study preparation for EM-M4. Our data did not find a benefit for students taking a comprehensive practice exam.

## LIMITATIONS

We acknowledge that this study has limitations. This was a single institution study, which limited our sample size and the amount of data available for analysis. Additionally, we were unable to distinguish between starting and completing a practice test, as only the raw data of score on the practice test is reported on the website. We did not have access to any specific student factors that may have impacted the scores, such as number of practice tests taken, etc. We assessed the data continuously as well as in binary and quaternary fashion. Other cutoffs may find a statistically significant difference. Due to a server catastrophe at www.saemtests.org, access to additional retrospective data and incorporation of other institutions was no longer available. It should be noted that we were looking at data from 2014–2017, which may not be transcribable to current SAEM tests due to their continuous program improvements.^10^ Prospective studies into this topic at other institutions are now possible as the practice tests have recently been transferred to a new website. In addition, the CDEM community is actively evaluating and assessing both their practice exams as well as both versions of the EM-M4 examination.

## CONCLUSION

The data obtained during this study carries implications for academic emergency medicine. Taking the available 2014–2017 SAEM practice tests as a studying modality did not appear to have a significant benefit in EM-M4 exam scores. Without knowledge of this lack of benefit, these practice tests were offered and promoted for EM-M4 examination preparation. If our results are generalizable, then we may be advising students to spend time and effort on an endeavor that we now suggest is low yield as compared to other study techniques.

## Figures and Tables

**Table 1:** Descriptive statistics for USMLE Step 2 CK and EM-M4 and the number of practice tests taken (N = 147).

Variable	Mean	SD	Minimum	Maximum	95% CI Lower	95% CI Upper
USMLE Step 2 CK	248.8	15.6	194	282	246.2	251.3
Practice Tests Taken	9.8	7.2	1	23	8.7	11.0
EM-M4*	83.1	6.9	62	96	81.9	84.2

* EM-M4 (Version 1) reported as percent score.

*USMLE*, United States Medical Licensing Examination; *CK*, clinical knowledge; *EM-M4*, emergency medicine fourth-year medical student; *SD*, standard deviation; *CI*, confidence interval.

**Table 2:** Pre- and post-analysis of covariance mean score adjustments (EM-M4 as dependent variable) based on number of practice tests taken and whether a comprehensive test was taken.

ANCOVA	Independent variables (groups)	N	ANCOVA mean	Adjusted ANCOVA mean
Number of practice tests taken	1–11 tests	87	82.1	82.3
	12–23 tests	60	84.4	84.1

*EM-M4*, emergency medicine fourth-year medical student; *ANCOVA*, analysis of covariance.

**Table 3:** Pre- and post-analysis of covariance mean score adjustments (EM-M4 as dependent variable) based on number of practice tests taken and whether a comprehensive test was taken.

ANCOVA	Independent variables (groups)	N	ANCOVA mean	Adjusted ANCOVA mean
Number of practice tests taken	1–6 tests	62	81.5	81.8
	7–12 tests	29	83.2	83.3
	13–18 tests	33	83.0	83.3
	19–23 tests	23	87.2	85.7

*EM-M4*, emergency medicine fourth-year medical student; *ANCOVA*, analysis of covariance.

**Table 4:** Pre- and post-analysis of covariance mean score adjustments (EM-M4 as dependent variable) based on the number of practice tests taken.

ANCOVA	Independent variables (groups)	N	ANCOVA mean	Adjusted ANCOVA mean
Number of practice tests taken	1	20	82.1	81.8
	2	11	80.9	82.2
	3	11	78.7	79.6
	4	6	80.9	80.8
	5	5	84.0	83.6
	6	9	83.3	84.0
	7	4	80.6	79.8
	8	5	82.0	83.0
	9	7	86.3	85.5
	10	4	84.4	85.7
	11	5	83.7	82.7
	12	4	80.1	81.7
	13	8	79.8	82.0
	14	7	86.9	87.7
	15	4	83.0	81.0
	16	9	83.3	83.3
	17	3	79.9	78.8
	18	2	85.0	85.2
	19	2	85.4	83.5
	20	5	88.3	86.2
	21	2	91.0	91.4
	22	3	87.8	86.4
	23	11	86.2	84.5

*EM-M4*, emergency medicine fourth-year medical student; *ANCOVA*, analysis of covariance.

**Table 5:** Pre- and post-analysis of covariance mean score adjustments (EM-M4 as dependent variable) based on whether a comprehensive test was taken.

ANCOVA	Independent variables (groups)	N	ANCOVA mean	Adjusted ANCOVA mean
Whether a comprehensive test was taken or not	Yes	82	83.5	83.2
	No	65	82.5	82.9

*EM-M4*, emergency medicine fourth-year medical student; *ANCOVA*, analysis of covariance.
